# Leptin sustains spontaneous remyelination in the adult central nervous system

**DOI:** 10.1038/srep40397

**Published:** 2017-01-16

**Authors:** Ken Matoba, Rieko Muramatsu, Toshihide Yamashita

**Affiliations:** 1 Department of Molecular Neuroscience, Graduate School of Medicine, Osaka University, Osaka 565-0871, Japan; 2 Core Research for Evolutional Science and Technology, Japan Science and Technology Agency, 5, Sanbancho, Chiyoda-ku, Tokyo 102-0075, Japan; 3 Precursory Research for Embryonic Science and Technology, Japan Science and Technology Agency, 5, Sanbancho, Chiyoda-ku, Tokyo 102-0075, Japan

## Abstract

Demyelination is a common feature of many central nervous system (CNS) diseases and is associated with neurological impairment. Demyelinated axons are spontaneously remyelinated depending on oligodendrocyte development, which mainly involves molecules expressed in the CNS environment. In this study, we found that leptin, a peripheral hormone secreted from adipocytes, promoted the proliferation of oligodendrocyte precursor cells (OPCs). Leptin increased the OPC proliferation via *in vitro* phosphorylation of extracellular signal regulated kinase (ERK); whereas leptin neutralization inhibited OPC proliferation and remyelination in a mouse model of toxin-induced demyelination. The OPC-specific leptin receptor long isoform (LepRb) deletion in mice inhibited both OPC proliferation and remyelination in the response to demyelination. Intrathecal leptin administration increased OPC proliferation. These results demonstrated a novel molecular mechanism by which leptin sustained OPC proliferation and remyelination in a pathological CNS.

Demyelination is a promising feature in many central nervous system (CNS) diseases^1^, such as multiple sclerosis. Because myelination ensures rapid propagation of action potentials by salutatory conduction and trophic support for axons in white matter tracts^2^, demyelination leads to impaired axonal homeostasis and consequent neurological deficits. Demyelinated axons are remyelinated spontaneously by the production of oligodendrocyte precursor cells (OPCs), which are adult stem cells that are widely distributed throughout CNS^3^. In response to a demyelinating injury, OPCs start to proliferate, migrate, and differentiate into myelin-expressing oligodendrocytes^4^. The process of remyelination is considered to be affected by the extracellular environment around the sites of demyelination; in addition, the mechanisms that regulate remyelination have been investigated by focusing on factors expressed by the CNS cells. In contrast, the role of factors derived from peripheral cells had not been fully clarified for remyelination.

Leptin is mainly secreted from adipose tissues and acts as a peripheral hormone that regulates food intake and increases energy expenditure^5^; these functions of leptin are tightly linked to the synaptic plasticity of neural circuits in the hypothalamus, where the long form of leptin receptors (LepRb) are expressed^6^. In this context, it is considered that peripheral leptin directly acts on the hypothalamus cells because of the absence of blood-brain barrier in this region^7^. This anatomical feature enables leptin influx into CNS; therefore, peripheral leptin may influence other CNS cells, such as oligodendrocytes, that express leptin receptors^8^. Although the function of leptin on oligodendrocyte development in adult CNS has not been clarified, previous reports indicate that leptin administration increased the myelin volume in mice spinal cord after injury, compared with that of control^8^. Therefore, we hypothesized that peripheral leptin is involved in oligodendrocyte development under pathological conditions in the adult CNS.

In this study, we showed that endogenous leptin sustained OPC proliferation in a mouse spinal cord after toxin-induced demyelination. Leptin treatment increased the number of OPCs by a mechanism that was dependent on *in vitro* extracellular signal regulated kinase (ERK) phosphorylation. Intrathecal administration of leptin-neutralizing antibodies and genetic ablation of LepRb inhibited OPC proliferation and remyelination in response to lysophosphatidylcholine (LPC)-induced demyelination. In addition, we found that intrathecal leptin treatment promoted OPC proliferation.

## Results

### Leptin promotes OPC proliferation

**Figure 1:**
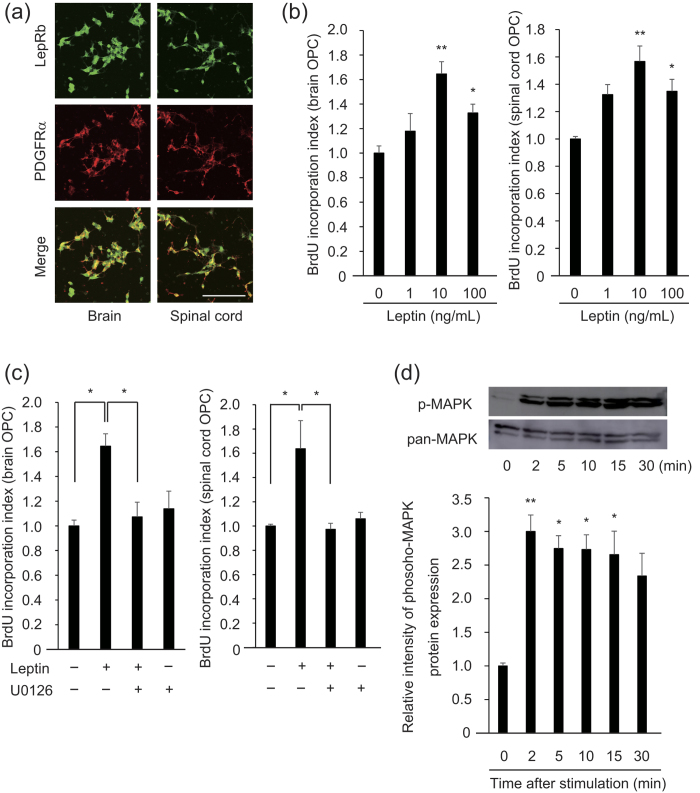
Leptin promotes OPC proliferation. (**a**) Representative image of cultured OPCs stained with antibodies against LepRb (green) and PDGFRα (red). Scale bar: 25 μm. (**b**) Relative BrdU incorporation into the OPC obtained from the brain (left graph) and spinal cord (right graph). Cells were treated with recombinant leptin for 48 h (n = 4). (Left graph) *P* = 0.005993 (control vs 10 ng/mL), 0.045616 (control vs 100 ng/mL), (Right graph) *P* = 0.004456 (control vs 10 ng/mL), 0.017859 (control vs 100 ng/mL). (**c**) Relative BrdU incorporation into the OPC after leptin stimulation (10 ng/ml) with U0126 (20 μM), a MEK inhibitor (n = 4 for brain OPCs, n = 3 for spinal cord OPCs). (Left graph) *P* = 0.019753 (control vs leptin), 0.039433 (leptin vs leptin + U0126), (Right graph) *P* = 0.045545 (control vs leptin), 0.04486 (leptin vs leptin + U0126). (**d**) Representative images of western blotting (upper panels) and quantitative analysis of ERK phosphorylation (lower graph) are shown. OPCs were treated with leptin (10 ng/ml) under indicated periods (n = 3). *P* = 0.006352 (2 min), 0.016571 (5 min), 0.017675 (10 min), 0.024100 (15 min), 0.081342 (30 min). *P < 0.05, **P < 0.01, ANOVA with Tukey’s post-hoc test.

To examine whether leptin promoted OPC proliferation, we first investigated whether OPC expressed leptin receptor. By immunocytochemistry, we confirmed the LepRb expression on PDGFRα-positive OPCs (Fig. 1a). We then investigated whether leptin stimulated OPC proliferation. Treatment with recombinant mouse leptin increased the ratio of Bromodeoxyuridine (BrdU) incorporation into the OPC obtained from brain and spinal cord (Fig. 1b). These results suggest that leptin promoted OPC proliferation. In immune cells, leptin receptors activate intracellular signaling, such as mitogen-activated protein kinase (MAPK)^9^, a well-known cell proliferation signaling^10^; therefore, we investigated the involvement of MAPK activation in leptin-mediated OPC proliferation. Treatment with MAPK kinase inhibitor U0126 abolished the leptin-mediated increase in BrdU incorporation (Fig. 1c), indicating that ERK phosphorylation was required for leptin-mediated OPC proliferation. We confirmed that leptin treatment enhanced ERK phosphorylation in OPC (Fig. 1d). These data suggested that leptin promoted OPC proliferation by a mechanism that was dependent on ERK phosphorylation.

### Leptin neutralization inhibits OPC proliferation and remyelination *in vivo*

**Figure 2:**
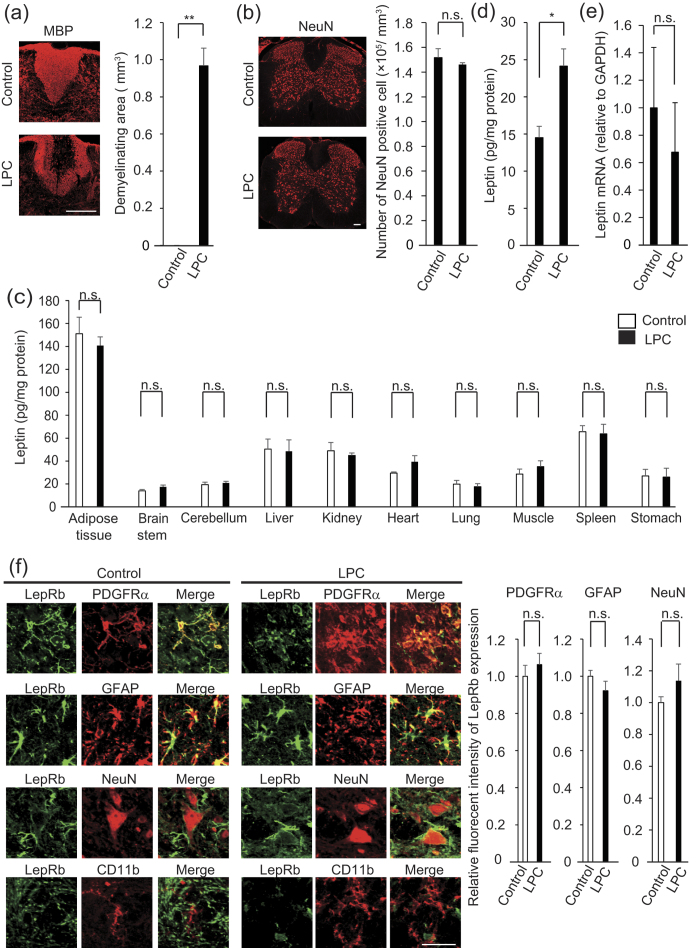
LPC injection does not enhance leptin expression in the CNS. (**a**) Representative images of MBP expression in a mouse spinal cord 14 days after LPC injection are shown; the graph shows quantification of the demyelinating area in the dorsal spinal cord (n = 3–4). *P* = 0.001542, Student’s *t*-test. (**b**) Representative images of NeuN expression in a mouse spinal cord 14 days after LPC injection; the graph shows quantification of the density of NeuN-positive cells in the spinal cord (n = 3). *P* = 0.299940, Student’s *t*-test, n.s. indicates no significant difference. (**c**) Quantification of leptin protein expression in indicated organs. Tissues were obtained from the mice 3 days after LPC injection (n = 3 for control, 4 for LPC injection). *P* = 0.318966 (adipose tissue), 0.10446 (brain stem), 0.332281 (cerebellum), 0.345245 (liver), 0.453104 (kidney), 0.098135 (heart), 0.335722 (lung), 0.236771 (muscle), 0.44662 (spleen), 0.465966 (stomach). Student’s *t*-test. n.s. indicates no significant difference. (**d**) Quantification of spinal cord leptin protein 3 days after LPC injection (n = 3 for control, 4 for LPC injection). *P* = 0.026865, Student’s *t*-test. (**e**) Quantification of spinal cord leptin mRNA 3 days after LPC injection (n = 6). *P* = 0.324930, Student’s *t*-test, n.s. indicates no significant difference. (**f**) Representative images of LepRb (green) expression in combination with PDGFRα, GFAP NeuN, and CD11b (red) in the mouse spinal cord with or without LPC injection. Spinal cord sections were obtained 3 days after LPC injection. Graph indicates the relative intensity of leptin protein expression in indicated cell type (n = 3). *P* = 0.287452 (PDGFRα), 0.181059 (GFAP), 0.199972 (NeuN), Student’s *t*-test, n.s. indicates no significant difference. *P < 0.05, **P < 0.01, error bars represent SEM. Scale bars; 100 μm for (**a** and **b)**, 25 μm for (**f)**.

To assess whether leptin promoted OPC proliferation *in vivo*, we used the toxin-induced demyelination model, in which the myelin structures were perturbed^11^,^12^,^13^ (Fig. 2a), but without neuronal damage (Fig. 2b). Leptin protein is expressed in adipose tissue abundantly^14^, which was confirmed in our model (Fig. 2c). We observed an increase in the levels of leptin around the demyelinating lesions after LPC injection (Fig. 2d); the level of leptin mRNA in the spinal cord was unchanged (Fig. 2e). In contrast, LepRb protein expression is detectable in PDGFRα-positive OPC, GFAP-positive astrocyte and NeuN-positive neuron in the spinal cord, but the intensity of LepRb immunoreactivity in these CNS cells was not changed in the response to LPC injection (Fig. 2f).

**Figure 3:**
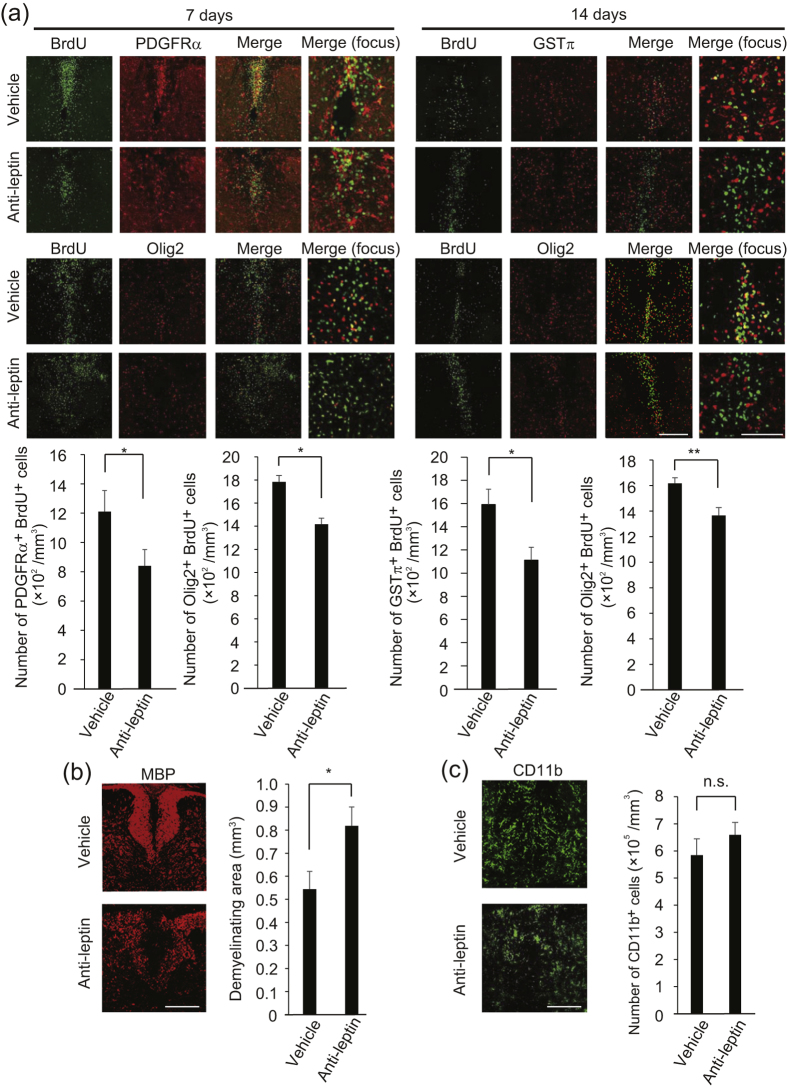
Endogenous leptin sustains spontaneous OPC proliferation. (**a**) Representative images of spinal cord sections, which were prepared 7 days (left panels) and 14 days (right panels) after LPC injection and double labeled for BrdU in combination with PDGFRα (upper panels), GSTπ (upper panels) and olig2 (lower panels). BrdU was administrated during 3–7 days after LPC injection; the graph shows quantification (n = 5–8). *P* = 0.042915 (PDGFRα and BrdU labeled cells), 0.013560 (Olig2 and BrdU labeled cells 7 days after injection), 0.012111 (GSTπ and BrdU labeled cells), 0.009797 (Olig2 and BrdU labeled cells 14 days after injection), Student’s *t*-test. (**b**) Representative spinal cord sections, which were prepared 14 days after LPC injection and stained with MBP are shown; the graph shows quantification (n = 6). *P* = 0.025243, Student’s *t*-test. (**c)** Representative images of spinal cord section, which were prepared 7 days after LPC injection and labeled for CD11b; the graph shows quantification (n = 4). *P* = 0.213763, Student’s *t*-test, n.s. indicates no significant difference. *P < 0.05, **P < 0.01, error bars represent SEM. Scale bar: 50 μm for high magnification images in **a**, 100 μm for others.

Next, we investigated whether leptin was involved in OPC proliferation after LPC injection. We started intrathecal administration of anti-leptin neutralizing antibodies at 3 days after LPC injection and evaluated the number of PDGFRα-positive OPCs around the demyelinating site. Immunohistochemical analysis revealed that, compared with the control, the mice treated with anti-leptin antibodies showed smaller numbers of BrdU and PDGFRα-double positive proliferating OPCs and GSTπ-positive mature oligodendrocytes in the dorsal spinal cord at 7 days and 14 days after LPC injection, respectively (Fig. 3a). To ask the possibility that endogenous leptin affect OPC differentiation, we compared the change of number of the cells labeled BrdU and PDGFRα 7 days after LPC injection and that of the cells labeled by BrdU and GSTπ 14 days after LPC injection. If endogenous leptin affects OPC differentiation, the number of cells labeled by BrdU and GSTπ 14 days after LPC injection is not comparative level with that of the cells labeled BrdU and PDGFRα 7 days after LPC injection. However, there is no significant difference between the increase of BrdU and PDGFRα-positive cells by anti-leptin antibodies treatment 7 days after LPC injection and that of BrdU and GSTπ-positive cells by anti-leptin treatment 14 days after LPC injection (P = 0.1531317, Fig. 3a), indicating that endogenous leptin does not affect OPC differentiation. Moreover, anti-leptin antibodies treatment did not affect the change of BrdU and Olig2-double positive cells number between 7 days and 14 days after LPC injection (P = 0.1974401, Fig. 3a), indicating that endogenous leptin does not affect the survival of oligodendrocyte lineage cells. Assessment of myelin formation by measurement of myelin basic protein (MBP)-positive area showed that, compared with the control, the mice treated with anti-leptin antibodies demonstrated larger demyelinating area in the spinal cord (Fig. 3b). Anti-leptin antibodies treatment did not affect the number of CD11b-positive microglia/macrophages around the site of LPC lesion (Fig. 3c). Therefore, these results indicated that leptin sustained the increase of OPC proliferation and subsequent remyelination.

### Leptin receptors are required for OPC proliferation

**Figure 4:**
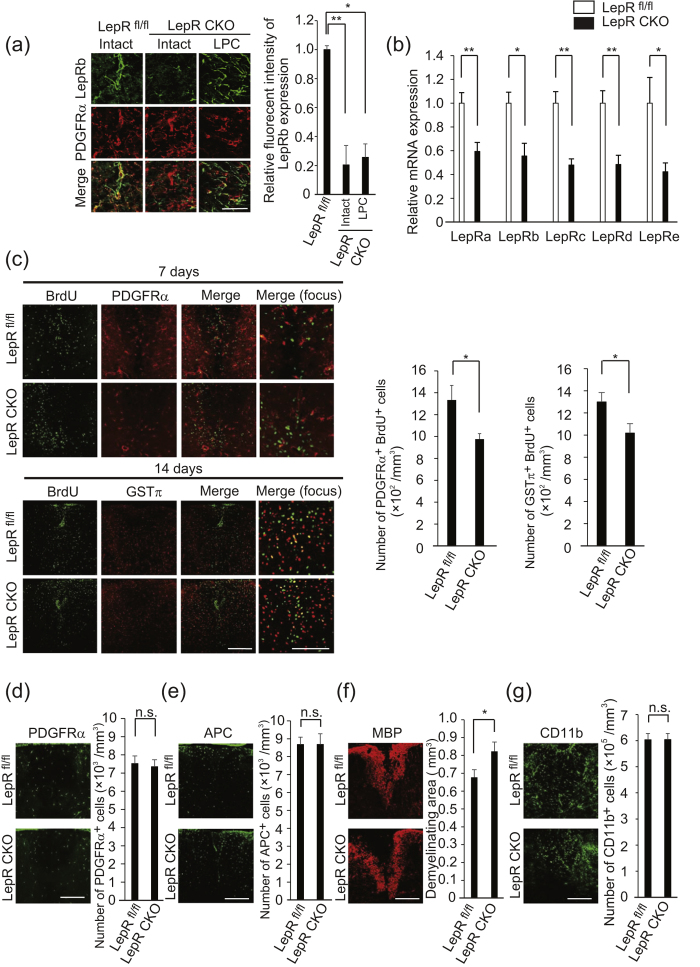
OPC expresses leptin receptors. (**a**) Representative images of spinal cord sections, which were double-labeled for LepRb (green) in combination with PDGFRα (red). Spinal cord sections were obtained 7 days after LPC injection; the graph shows quantification (n = 3). *P* = 0.007573 (LepRb flox vs intact CKO), 0.0108779 (LepRb flox vs LPC CKO), ANOVA with Tukey’s post-hoc test. (**b**) Relative expression of leptin receptors mRNA in PDGFRα-positive OPC obtained from the brain of *PDGFR*α-creERT:: *Lepr* flox/flox mice and +/+::*Lepr* flox/flox mice (n = 5,6). *P* = 0.005878 (LepRa), 0.010306 (LepRb), 0.001535 (LepRc), 0.003169 (LepRd), 0.030459 (LepRe), Student’s *t*-test. (**c)** Representative images of spinal cord sections which were double labeled for BrdU in combination with PDGFRα (left panels) and GSTπ (right panels). Sections were prepared 7 days (left panels) and 14 days (right panels) after LPC injection. BrdU was administrated during 3–7 days after LPC injection; the graph shows quantification (n = 5–8). *P* = 0.029791(PDGFRα and BrdU labeled cells), 0.028870 (GSTπ and BrdU labeled cells), Student’s *t*-test. (**d)** Representative images of PDGFRα expression in the intact spinal cord of *PDGFR*α-creERT:: *Lepr* flox/flox mice and +/+::*Lepr* flox/flox mice; the graph shows quantification (n = 3–4). *P* = 0.404999, Student’s *t*-test, n.s. indicates no significant difference. (**e**) Representative images of APC expression in the intact spinal cord of *PDGFR*α-creERT:: *Lepr* flox/flox mice and +/+::*Lepr* flox/flox mice; the graph shows quantification (n = 3). *P* = 0.495667, Student’s *t*-test, n.s. indicates no significant difference. (**f**) Representative spinal cord section of *PDGFR*α-creERT:: *Lepr* flox/flox mice, which were prepared 14 days after LPC injection and stained with MBP; the graph shows quantification of the demyelinating area in the dorsal spinal cord (n = 7 for control, 10 for CKO). *P* = 0.030688, Student’s *t*-test. (**g**) Representative spinal cord sections which were labeled for CD11b. Sections were prepared 7 days after LPC injection. The graph shows quantification (n = 3). *P* = 0.493264, Student’s *t*-test, n.s. indicates no significant difference. *P < 0.05, **P < 0.01, error bars represent SEM. Scale bars: 25 μm for (**a)**, 50 μm for high magnification images in (**c)**, 100 μm for others.

Next, we probed whether leptin-mediated OPC proliferation depended on leptin receptor expression in OPCs. Immunohistochemical analysis of the spinal cord revealed the expression of LepRb in PDGFRα-positive OPCs of intact adult mice (Fig. 4a). Real time PCR analysis showed that OPC expressed all the subtypes of leptin receptors mRNA, including *LepRb*, the main receptor responsible for leptin signaling^15^ (Fig. 4b).

Because we observed the LepRb expression on astrocyte and neuron as well as on OPCs (Fig. 2f), we generated conditional knockout mouse with *Lepr* knockdown in the PDGFRα-positive OPC to investigate the specific the impact for leptin receptors on OPCs. Immunohistochemical analysis confirmed that tamoxifen-inducible Cre-mediated recombination reduced LepRb protein expression in PDGFRα-positive cells in the conditional knockout mice (*Pdgfr*α-Cre/−:: *Lepr* flox/flox) compare with control littermate (−/−:: *Lepr* flox/flox mice) (Fig. 4a). RT-PCR confirmed decreased expression of all types of *Leptin receptors* mRNA in PDGFRα-positive cells of conditional knockout mice, compared with control littermates (Fig. 4b).

We then conducted LPC injection into the spinal cord of the conditional knockout mice and performed histological analysis to count the number of proliferating OPCs and mature oligodendrocytes in the spinal cord. The number of BrdU and PDGFRα-double positive cells and GSTπ-positive cells in the spinal cord of conditional knockout mice was smaller than that of the control at 7 days and 14 days after LPC injection, respectively (Fig. 4c). The change of BrdU and PDGFRα-double positive cells in conditional knockout mice compared with that in control is comparative to that of BrdU and GSTπ-double positive cells in conditional knockout mice compared with that in control 14 days after LPC injection (Fig. 4c). There were no significant differences in PDGFRα-positive cells or APC-positive cells between the conditional knockout mice and the control mice under intact conditions (Fig. 4d and e). These data indicate that leptin receptor in OPC is not involved in OPC differentiation. We confirmed that these observations relied on myelin formation by MBP staining at 14 days after LPC injection (Fig. 4f). The number of CD11b-positive cells was not changed between the groups (Fig. 4g). These data suggest that LepRb in OPCs is required for OPC proliferation and remyelination.

**Figure 5:**
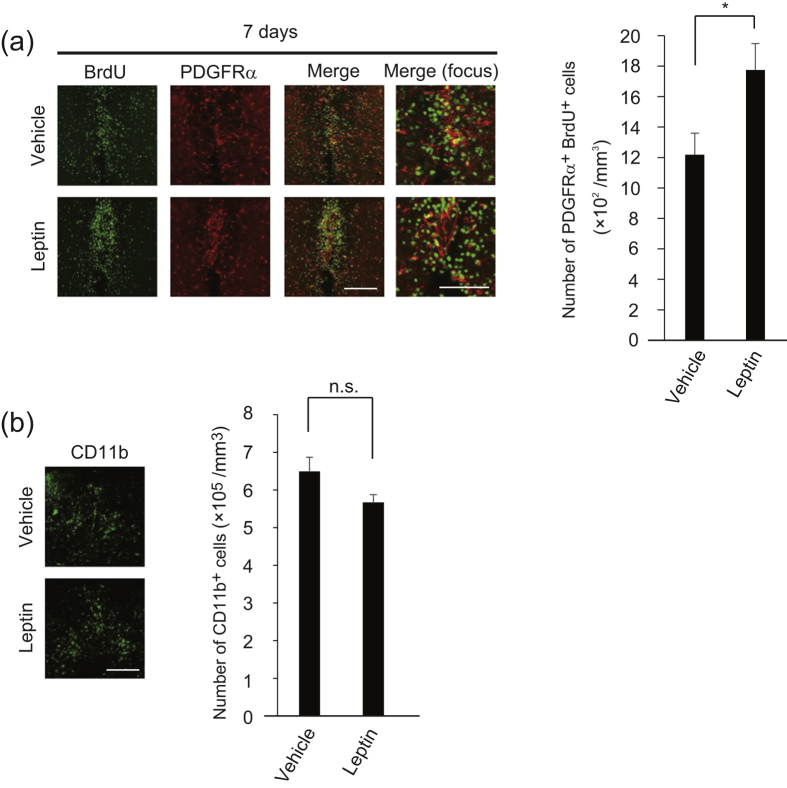
Leptin promotes OPC proliferation. (**a**) Representative images of spinal cord sections which were double-labeled for BrdU with PDGFRα. BrdU was administrated during 3–7 days after LPC injection and sections were prepared 7 days after LPC injection; the graphs show quantification (n = 7). *P* = 0.020898, Student’s *t*-test. (**b)** Representative images of spinal cord sections which were labeled with CD11b. Sections were prepared 7 days after LPC injection; the graph shows quantification (n = 3 for control, 4 for leptin treatment). *P* = 0.091678, Student’s *t*-test. *P < 0.05, error bars represent SEM Scale bars: 50 μm for high magnification images in (**a)**, 100 μm for others.

Given that OPC proliferation was mediated by endogenous leptin, we investigated the possibility that leptin treatment exerted therapeutic actions on demyelination. Intrathecal administration of recombinant mouse leptin increased the number of BrdU and PDGFRα-double positive cells in the spinal cord in the spinal cord 7 days after LPC injection (Fig. 5a). The number of CD11b-positive cells around the lesion was not changed with or without leptin treatment (Fig. 5b). These data indicated that exogenous leptin treatment may enhance OPC proliferation.

## Discussion

We found that leptin sustained OPC proliferation and contributed to remyelination in the adult CNS. The mechanism of remyelination has been investigated by focusing on the molecules in the CNS microenvironment; therefore, our findings provided the possibility that in pathological states of the CNS, the peripheral environment may also contribute to remyelination. In this context, we found that leptin promoted OPC proliferation and contributed to remyelination. The association of leptin with oligodendrocyte development has been pointed out by reports that leptin-deficient *ob/ob* mice brain had a significantly lower amount of myelin compared with that of control (+/+) mice^16^. During brain development, leptin receptors are not expressed in OPC, but are detected in the late-phase oligodendrocyte progenitors^17^,^18^. Therefore, leptin-mediated myelination may be strongly supported by the promotion of the late phase of oligodendrocyte development, such as differentiation of oligodendrocyte progenitors into mature oligodendrocytes and/or increase in myelin-associated protein expression. Meanwhile, we detected that OPCs expressed leptin receptors that contributed to OPC proliferation in response to demyelination in a pathological adult CNS. Therefore, the function of leptin on oligodendrocyte development may differ between normal development and pathological conditions; one possible mechanism that may explain this difference is the change in leptin receptor expression. It was reported that leptin receptor expression in rodents was increased by several pathological stimuli, such as hypoxia^19^, injury^8^, and cytokines (IGF-1)^20^. Additional experiments that will clarify the changes in leptin receptor expression under pathological CNS conditions may enable further understanding of the role of leptin in oligodendrocyte development after CNS damage.

We observed the LepRb expression on astrocyte and neuron as well as on OPCs. This observation raises the possibility that leptin act on non-OPC cells resulting in increase of the OPC proliferating factor production and contribute to OPC proliferation. At present, it is not clarified that leptin-stimulated astrocyte and neuron increase the production of well-known OPC proliferation factor. However, there is no significant difference between the increase of BrdU and PDGFRα-positive cells by anti-leptin antibodies treatment after LPC injection and that of BrdU and PDGFRα-positive cells by LepRb deletion after LPC injection (Pint = 0.442048), highlighting that leptin have direct action to OPC.

Our observations suggested that leptin treatment promoted CNS remyelination, which is a regenerative process that is associated with recovery from neurological deficits. Therefore, we speculated that leptin treatment may be beneficial for treating demyelinating diseases. However, we should note that leptin has a proinflammatory immune response^21^. A previous report suggested that leptin reduces the number of immunosuppressive regulatory T cells in an experimental autoimmune encephalomyelitis (EAE), an animal model of multiple sclerosis^22^. Moreover, leptin neutralization inhibits T cell proliferation and changes the T cell profile, which is associated with improvement in clinical score and prevention of disease progression in EAE^23^. Therefore, leptin therapy for CNS pathologies may not guarantee the absence of detrimental effects.

Investigation on leptin-mediated OPC-specific signal transduction may develop the leptin-associated remyelination method. Among the various intracellular signal transduction processes mediated by leptin, we focused on the involvement of ERK activation, which previously pointed out the role of myelination^24^. However, because ERK is almost universally expressed in many cells, we cannot indicate OPC-specific leptin-mediated signaling. OPC has cell type-specific signal transduction^25^; therefore, if we identify OPC-specific signal transduction by leptin, these mechanisms will enhance the understanding of the molecular biology of leptin and may enable development of therapies for demyelinating diseases.

## Methods

### Mice

This study was approved by the institutional committee of Osaka University. C57BL/6 J mice were obtained from Charles River Japan or Japan SLC. B6N.Cg-Tg (Pdgfra-cre/ERT) 467Dbe/J (stock no. 018280) and B6.129P2-Leprtm1Rck/J (stock no. 008327) were purchased from the Jackson Laboratory. The experiments were performed in accordance with the Guide for the Care and Use of Laboratory Animals of the Graduate School of Medicine Osaka University (no. 24-067-055).

### Primary culture of OPC and BrdU incorporation assay

Primary culture of OPC was isolated from brain and spinal cord of C57BL/6 J mice at postnatal day 1 (ref. 26). Tissues were dissected in ice-cold phosphate-buffered saline (PBS) and dissociated into single-cell suspensions using 0.25% trypsin solution. Single cell suspension was treated with anti-PDGFRα antibodies-conjugated microbeads (Miltenyi-Biotec). Isolated cells (OPCs) were plated on poly-L-lysine–coated 96-well plates (Greiner Bio-One) at a density of 1.5 × 10^4^ cells/ well. The cells were maintained at 37 °C with 5% CO_2_ and cultured in Dulbecco’s modified Eagles medium (DMEM) supplemented with 4 mM L-glutamine, 1 mM sodium pyruvate (Sigma), 0.1% bovine serum albumin (BSA, Sigma), 50 μg/ml apo-transferrin (Sigma), 5 μg/ml insulin (Sigma), 30 nM sodium selenite (Sigma), 10 nM biotin (Sigma), 10 nM hydrocortisone (Sigma), 10 ng/ml platelet-derived growth factor (PDGF)-AA (PeproTech), and 10 ng/ml basic-fibroblast growth factor (FGF) (PeproTech). The ratio of BrdU incorporation was evaluated by using the Cell Proliferation ELISA and BrdU (colorimetric) kit (Roche). Cells were incubated 24 h after BrdU addition. To inhibit MAPK kinase, cells were pre-treated with 20 μM of U0126 (9903, Cell Signaling Technology) for 15 min before the start of recombinant leptin treatment.

### Immunocytochemistry

Cultures were fixed with 4% paraformaldehyde (PFA) in PBS for 30 min at room temperature. Cells were permeabilized with PBS containing 0.3% Triton X-100 and 10% goat serum (Sigma), followed by overnight treatment with primary antibodies at 4 °C. Cells were then incubated with fluorescent-labeled secondary antibody for 1 h at room temperature. The following antibodies were used: rat anti-mouse PDGFRα (1:500, 558774, BD Biosciences); chicken anti-rat LepRb (1:100, CH14104, Neuromics); Alexa Fluor 488-conjugated donkey antibody against chicken IgG and Alexa Fluor 568-conjugated goat antibody against rat IgG. Immunofluorescence images were captured with an Olympus BX60 fluorescence microscope equipped with a cooled CCD camera (DP80; Olympus).

### Western Blotting

OPCs were plated at a density 2.7 × 10^5^ cells/ml and were treated with 10 ng/ml of recombinant mouse leptin. After culturing, the cells were collected in lysis buffer (50 mM Tris-HCl at pH 7.4, 150 mM NaCl, 1% Triton-X 100, and 50 μM EDTA) containing a cocktail protease inhibitors (Roche). The lysates were clarified by centrifugation at 15,900 × g at 4 °C for 10 min, and the supernatants were collected and normalized for protein concentration. Proteins were separated by 9% SDS-PAGE, and then transferred onto polyvinylidene fluoride membranes (Immobilon-P, Millipore). The membranes were incubated with PBS containing 5% skim milk and 0.05% Tween 20 for 1 h at room temperature following incubation with primary antibodies. Antibody binding was detected by an electro-generated chemiluminescence system (Amersham Imager 600, GE Healthcare). The intensity of protein bands was quantified using ImageJ software (NIH). The following antibodies were used: anti-phospho-p44/p42 MAPK (ERK1/2) (Thr202/Tyr204) (1:1000, 9101 S, Cell Signaling Technology); anti-p44/42 MAPK (ERK 1/2) (1:1000, 9102 S, Cell Signaling Technology); and horseradish peroxide (HRP-conjugated anti-rabbit IgG (1:5000, eB182, TrueBlot).

### Surgical procedure

Adult female mice at 7–8 weeks of age underwent laminectomy at Th11/Th12; 2 μl of 10 mg/ml lysophosphatidylcholine (LPC, L1381, Sigma Aldrich) dissolved in PBS was injected into the midline dorsal column at a depth of 0.5 mm^13^. For administration of recombinant mouse leptin (Sigma Aldrich) or leptin neutralizing antibodies (AF498, R&D System), a cannula from Alzet osmotic pump (model No. 1002 or 1007D, Alzet Corp.) was placed at the thoracic spinal cord under the dura 3 days after LPC injection. The pump was filled with vehicle solution (PBS); recombinant mouse leptin (12 μg/kg body weight per day) or leptin neutralizing antibodies (10 μg/kg of body weight per day) were administered subcutaneously on the back.

### BrdU labeling

BrdU (1 mg/ml) was added to the drinking water during 3–7 days after LPC injection. The water bottles containing BrdU was protected from light and changed every 3 days during labeling periods.

### Immunohistochemistry

At 0, 3, 7 and 14 days after LPC injection, mice were transcardially perfused with 4% PFA in PBS. Spinal cords were post-fixed with 4% PFA in PBS for 1 h (for PDGFRα staining) or overnight at 4 °C; and equilibrated in 15% sucrose-PBS for 24 h, then in 30% sucrose-PBS until subsidence at 4 °C. Tissues were embedded in an optimal cutting temperature compound (Tissue-Tek); transverse sections were cut at 30-μm thickness (spaced 300 μm apart including lesion epicenter) on a cryostat and mounted on Matsunami adhesive silane-coated slides (Matsunami Glass). For detecting BrdU-labeled cells, the sections were pretreated with boiling citrate buffer (pH 6.0) in a microwave oven for 10 min and washed in 0.1 M borate buffer (pH 8.5) for 10 min. The sections were permeabilized with PBS containing 0.3% Triton X-100 and 10% goat serum (Sigma) (for PDGFRα staining) or with PBS containing 0.1% Triton X-100 and 5% BSA for 30 min at room temperature. The sections were then incubated with primary antibodies overnight at 4 °C, followed by incubation with fluorescent-labeled secondary antibody for 1 h at room temperature. Rat anti-mouse MBP (1:100, ab7349, Abcam), chicken anti-rat LepRb (1:100, CH14104, Neuromics), rabbit anti-mouse GSTπ (1:300, 312, MBL), rabbit anti-human Olig2 (1:500, 18953, IBL), rat anti-mouse CD11b (1:100, 550282, BD Pharmingen), goat anti-mouse PDGFRα (1:100, AF1062, R&D System), rat anti-mouse BrdU (1:100, 0BT0030G, AbD Serotec), mouse anti-mouse GFAP (1:100, G3893, Sigma), mouse anti-mouse APC (1:50, OP80, Calbiochem) and mouse anti-mouse NeuN (1:100, MAB377, Chemicon) were used as primary antibodies. Alexa Fluor 488-conjugated goat antibody against rat IgG, donkey antibody against chicken IgG, donkey antibody against rat IgG, goat antibody against mouse IgG and Alexa Fluor 568-conjugated goat antibody against rat IgG, goat antibody against mouse IgG, goat antibody against rabbit IgG, donkey antibody against goat IgG were used as secondary antibodies. Immunofluorescence images were captured with an Olympus Fluoview FV1200 microscope (Olympus, Tokyo, Japan). For quantitation, each 8 to10 sections per mouse were collected and were used for calculation of the average of cell number and myelinating area.

### Conditional knockout mice

The OPC-specific leptin receptor deletion mice were obtained by crossing the *Lepr* flox mice with the *Pdgfr*α-cre/ERT mice. Cre recombination in the generated mice was induced by administering tamoxifen (75 mg/kg, i.p.; Sigma-Aldrich) on each of the 11 consecutive days. To assess the efficiency of *Lepr* deletion at mRNA level, OPC was obtained from the brains of Cre/−::flox/flox mice (conditional knockout mice) and −/−::flox/flox mice (control littermates) using PDGFRα-specific antibody-coated magnetic beads (Milteny-Biotech). The relative *Lepr* expressions in the isolated OPCs were assessed by real time PCR. To confirm reduction of leptin receptor protein expression in OPCs, spinal cord sections were obtained from conditional knockout mice and control littermates. The sections were immunostained with antibodies against LepRb and PDGFRα, and the fluorescence intensity of LepRb in PDGFRα-positive cells was measured by ImageJ software. The relative fluorescent intensity of LepRb was calculated by the using of value obtained from control littermates.

### Quantitative RT-PCR

RNA was isolated using TRIzol reagent (Invitrogen) and was purified using a RNA Clean & Concentrator-5 column (Zymo Research). cDNA synthesis was performed on 2 μg of total RNA using High Capacity cDNA Reverse Transcription Kit (Applied Biosystems). Samples for the Taqman assays consisted of 1× final concentration of Taqman gene expression master mix (Applied Biosystems), 500 nM of gene-specific primers, and 250 nM of Taqman probe. PCR conditions included one cycle at 50 °C for 2 min and 95 °C for 10 min; and 40 cycles at 95 °C for 15 seconds and at 60 °C for 1 min. A melting analysis was carried out after PCR to monitor the specificity of amplification. Relative mRNA expression level was detected by ABI ViiA7 real-time PCR system (Applied Biosystems) and was normalized with the glyceraldehyde-3-phosphate dehydrogenase (GAPDH). The following primers were used: *Lepra* forward, GAAGTCTCTCATGACCACTACAGATGA; *Lepra* reverse, TTGTTTCCCTCCATCAAAATGTAA; *Lepra* probe, FAM-CCCAATCTACCAACTTCCCAACAGTCCA-TAMRA; *Leprb* forward, GCTCTTCTGATGTATTTGGAAATC; *Leprb* reverse, ACCTGATATTGAAGCGGAAATGG; *Leprb* probe, FAM-CCTCTTCTTCTGGAGCCTGAACCCATTTC-TAMRA, *Leprc* forward, TCGACAAGCAGCAGAATGAC; *Leprc* reverse, CAGTGACCTTTTGGAAATTCAGTC; *Leprc* probe, FAM-TTGTGTCCTACTGCTCGGAA-TAMRA; *Leprd* forward, GGAAGGAGTTGGAAAACCAAAGA; *Leprd* reverse,TCCTTTTGGAAATTCAGTCCTTGTG; *Leprd* probe, FAM-GCAGCAGAATGACGCAGGGC-TAMRA; *Lepre* forward, GCAGCTGTGTCATCCTTTCC; *Lepre* reverse, GTACAGTACACATACCGTGG; *Lepre* probe, FAM-GTGGCTTAGAATTCCCTCGA-TAMRA; *GAPDH* forward, TGTGTCCGTCGTGGATCTGA; *GAPDH* reverse, CCTGCTTCACCACCTTCTTGA; *GAPDH* probe, FAM-CCGCCTGGAGAAACCTGCCAAGTATG-TAMRA.

### Enzyme-linked immunosorbent assay (ELISA)

Mice were transcardially perfused with 20 ml PBS and tissues were isolated from the mice and were homogenized in lysis buffer (160 mM KCl; 25 mM HEPES; 1% Triton-X 100; and a complete cocktail of protease inhibitors [Roche]). After centrifugation at 15,900 × g for 20 min at 4 °C, the supernatant was collected and normalized for protein concentration using standard bicinchoninic acid (BCA) assay. Leptin concentrations in the samples were measured with a specific ELISA (EZML-82K, Linco Research). To measure the leptin protein level around the demyelinating lesion, spinal cord tissues (Th10–12) were collected 3 days after LPC injection and were used for sample preparation for ELISA assay as described above.

### Statistical analysis

Data were presented as mean ± SEM. Statistical significance between groups was examined using either unpaired Student’s *t*-test or one-way ANOVA, followed by Tukey–Kramer test. The difference of cell number was compared with single regression analysis; *P* < 0.05 was considered to be significant.

## Additional Information

**How to cite this article:** Matoba, K. *et al*. Leptin sustains spontaneous remyelination in the adult central nervous system. *Sci. Rep.* **7**, 40397; doi: 10.1038/srep40397 (2017).

**Publisher's note:** Springer Nature remains neutral with regard to jurisdictional claims in published maps and institutional affiliations.
